# Trust in researchers and willingness to engage in cancer research

**DOI:** 10.1093/oncolo/oyag170

**Published:** 2026-04-30

**Authors:** Kalyani Sonawane, Alexander Alekseyenko, Gayenell S Magwood, Shikhar Mehrotra, Gayathri R Devi, Marvella E Ford

**Affiliations:** Department of Public Health Sciences, College of Medicine, Medical University of South Carolina, Charleston, SC 29425, United States; MUSC Hollings Cancer Center, Charleston, SC 29425, United States; Department of Public Health Sciences, College of Medicine, Medical University of South Carolina, Charleston, SC 29425, United States; MUSC Hollings Cancer Center, Charleston, SC 29425, United States; Department of Biobehavioral Health and Nursing Science, College of Nursing, University of South Carolina, Columbia, SC 29208, United States; MUSC Hollings Cancer Center, Charleston, SC 29425, United States; Department of Surgery, College of Medicine, Medical University of South Carolina, Charleston, SC 29425, United States; MUSC Hollings Cancer Center, Charleston, SC 29425, United States; Department of Surgery, College of Medicine, Medical University of South Carolina, Charleston, SC 29425, United States; Department of Public Health Sciences, College of Medicine, Medical University of South Carolina, Charleston, SC 29425, United States; MUSC Hollings Cancer Center, Charleston, SC 29425, United States

**Keywords:** trust, research participation, biobanking, clinical trials

## Abstract

**Background:**

Trust in medical scientists shapes public engagement in health and biomedical research, yet its influence on cancer research participation is not well understood. This study assessed trust in researchers and examined its relationship with willingness to engage in diverse cancer research activities.

**Methods:**

Cross-sectional analyses of a 2023 statewide survey of US adults evaluated Trust in Medical Researcher Scale (TMRS) scores (range 0-48) and willingness to participate in cancer research activities (research studies, biobanking, and data-sharing). Associations between trust and participation were examined using descriptive statistics and adjusted logistic regression models.

**Results:**

Among 1780 respondents, the mean TMRS score was 27.3 ± 9.3, with most reporting moderate trust. Willingness to participate in cancer research varied across activity types. Low trust was consistently associated with reduced willingness across all activity types.

**Conclusions:**

Limited trust in researchers represents a significant barrier to participation in cancer research.

Recent surveys in the United States (US) reveal a sharp decline in public trust in medical scientists and growing skepticism toward health institutions, including reduced confidence in cancer-related information from government agencies.^1^^,^^2^ Cancer has become one of the most frequent targets of health misinformation, with false “cures,” misleading prevention claims, and conspiracy narratives circulating widely across digital platforms. Compounding this issue, a rising number of individuals are engaging with cancer misinformation and harmful content, which is undermining public trust.^3^

Trust in medical scientists is a key driver of engagement in public health and biomedical research. Despite this, little is known about how trust in researchers shapes individuals’ willingness to participate in cancer research. This study fills that gap by quantifying trust in cancer researchers among US adults and evaluating its relationship with willingness to engage in diverse cancer research activities.

## Methods

This cross-sectional study analyzed data from a statewide survey of US adults aged ≥18 years conducted between January and December 2023. Individuals living in South Carolina were recruited using random digit-dialing, and data were collected via Computer-Assisted Telephone Interviews (CATI), which included sociodemographic information and a validated 12-item *Trust in Medical Researcher* Scale (TMRS; score range 0-48, with lower scores indicating lower trust) (Table S1).^4^ The survey also assessed willingness to engage in a cancer research study, provide biospecimens, and share medical or laboratory records over the next three months (Table S2). The study was deemed exempt by the Medical University of South Carolina IRB due to de-identified data. Descriptive statistics summarize participant characteristics, mean TMRS scores, and trust levels. Associations between trust level and willingness to engage in cancer research were examined using logistic regression models adjusted for sociodemographic factors. Statistical significance set at *P *< .05. All analyses were performed using SAS (Cary, NC).

## Results

The final analytic sample consisted of 1780 respondents with non-missing responses for TMRS and willingness to engage in cancer research. Participants were predominantly adults aged 65 years or older (24.5%), non-Hispanic (94.5%), White (62.7%), female (50.7%), with a high school education (22.8%), and residing in metropolitan areas (87.3%). The mean overall trust score in cancer researchers was 27.3 (SD = 9.3) with low (≤16; 3.9%), moderate (17-32; 81.5%), and high (≥33; 14.4%) levels of trust.

**Figure 1:**
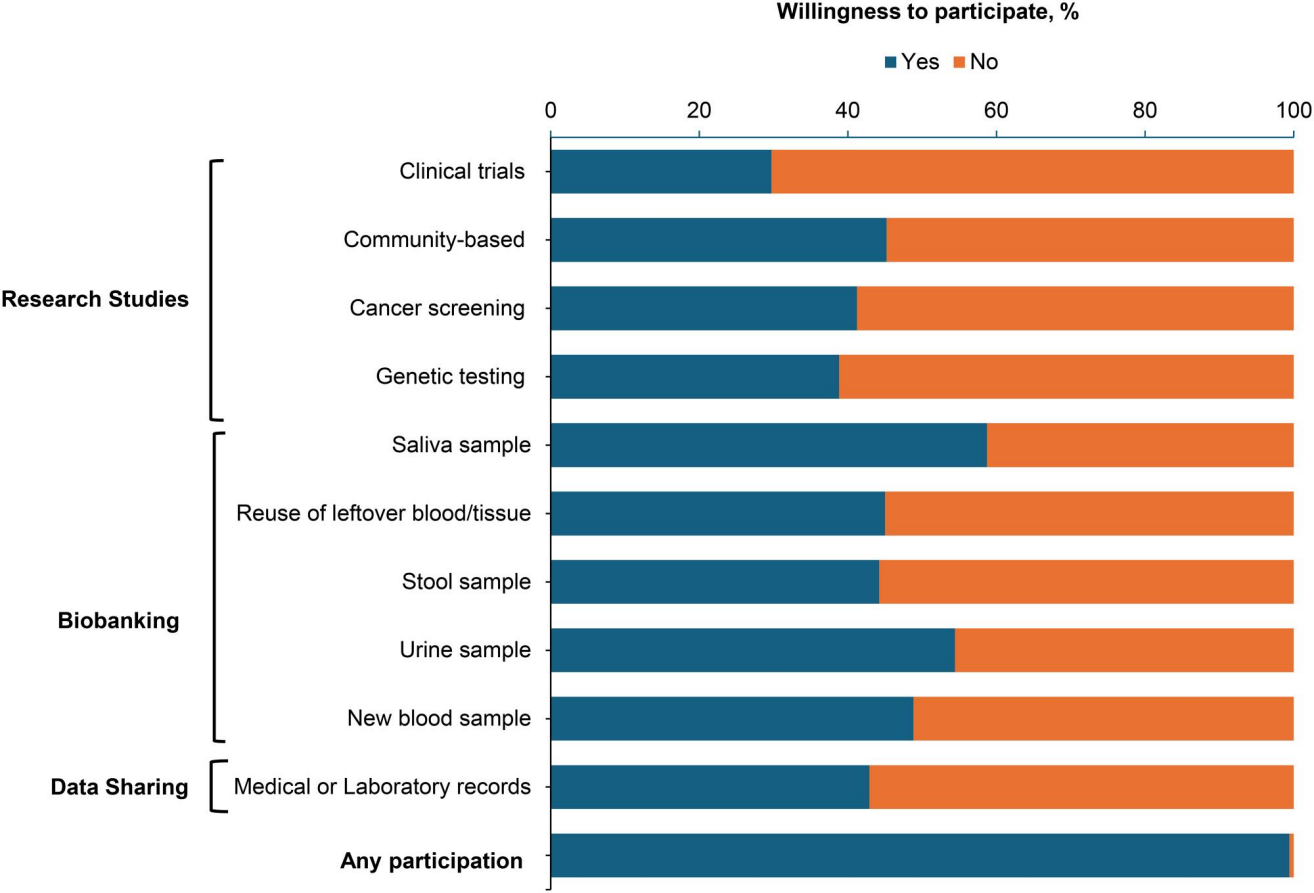
Willingness to engage in cancer research among participants by type of research activity. This figure illustrates willingness to engage in cancer research among 1780 survey respondents. Participants were asked about their willingness to engage in public health and biomedical research activities, including research studies (clinical trials, community-based studies, cancer screening, and genetic testing); providing biospecimens (saliva, blood, tissue, fluid, stool, and urine); and sharing medical or laboratory records in the next three months if approached by a medical research team. Bars represent the proportion of participants who indicated willingness (responded “*Yes*”) or a lack of willingness (responded “*No*”) to engage in cancer research.

Willingness to participate in cancer research varied across activity types (Figure 1). For research studies, fewer than half of respondents reported willingness to participate, including clinical trials (29.7%), genetic testing (38.8%), cancer screening studies (41.2%), and community-based research (45.2%). Biobanking activities showed slightly higher willingness, although only saliva (58.7%) and urine sample donation (54.4%) exceeded 50%, while reuse of leftover blood or tissue (45.0%), stool samples (44.2%), and new blood samples (48.8%) remained below this threshold. Willingness to share medical or laboratory records was similarly low at 42.9%. Overall, most participants were willing to participate in at least one type of activity.

Across all research activity types, lower levels of trust in researchers were consistently associated with reduced willingness to participate in cancer research activities (Table 1). Compared with individuals reporting high trust, those with moderate trust had significantly lower odds of participating in research studies, including clinical trials (adjusted odds ratio [AOR] = 0.52, 95% CI, 0.39-0.69), community-based studies (0.45, 0.34-0.60), cancer screening studies (0.36, 0.27-0.48), and genetic testing (0.44, 0.33-0.58). Similar patterns were observed for biobanking activities, where moderate trust was associated with substantially lower willingness to provide saliva samples (0.23, 0.17-0.34), urine samples (0.42, 0.31-0.58), new blood samples (0.38, 0.28-0.51), and other biospecimens. Individuals with low trust demonstrated even lower odds of participation across most activities, particularly for clinical trials (0.44, 0.24-0.81), reuse of leftover blood or tissue (0.51, 0.29-0.89), and sharing medical or laboratory records (0.21, 0.12-0.39). Overall, participants with low trust in researchers had lower odds of engaging in any cancer research (0.11, 0.02-0.71) than those with high trust.

**Table 1:** Trust in researchers and its association with willingness to engage in cancer research.

Activity type	Willingness to engage in cancer research		
	Adjusted odds ratio (95% confidence interval)^a^		
	High trust	Moderate trust	Low trust
**Research studies**			
** Clinical trials**	Ref	0.52 (0.39-0.69)	0.44 (0.24-0.81)
** Community-based**	Ref	0.45 (0.34-0.60)	0.46 (0.27-0.80)
** Cancer screening**	Ref	0.36 (0.27-0.48)	0.48 (0.28-0.84)
** Genetic testing**	Ref	0.44 (0.33-0.58)	0.58 (0.34-1.00)
**Biobanking**			
** Saliva sample**	Ref	0.23 (0.17-0.34)	0.29 (0.16-0.54)
** Reuse of leftover blood/tissue**	Ref	0.45 (0.34-0.60)	0.51 (0.29-0.89)
** Stool sample**	Ref	0.57 (0.43-0.75)	0.62 (0.35-1.07)
** Urine sample**	Ref	0.42 (0.31-0.58)	0.43 (0.25-0.76)
** New blood sample**	Ref	0.38 (0.28-0.51)	0.36 (0.20-0.62)
**Data sharing**			
** Medical or laboratory records**	Ref	0.39 (0.29-0.52)	0.21 (0.12-0.39)
** Any participation**	Ref	2.9 (0.50-16.3)	0.11 (0.02-0.71)

a Models were adjusted for age, sex, race, ethnicity, education, and area of residence. Willingness to engage in research was modeled separately for each type of engagement. Information on race (21), ethnicity (1) and sex (8) was missing for some participants. Trust was measured using the Trust in Medical Researcher Scale (TMRS); trust levels included low (TMRS score ≤16), moderate (TMRS score 17-32), and high trust (TMRS score ≥33).

## Discussion

This study offers critical insights into trust in researchers in the general US population and its association with participation in public health and biomedical research, with particular attention to engagement in cancer research. Only about 14% of individuals expressed high trust, highlighting a barrier to engagement in cancer research. Concerningly, aside from providing saliva or urine samples, fewer than half of respondents were willing to engage in most cancer research studies. Within the broader landscape of declining trust in science, this finding illustrates how anti-science movements and the spread of misinformation are distorting public perceptions of medical scientists and undermining efforts in cancer prevention, treatment, and prognosis research.

Individuals with moderate or low trust were consistently less willing to engage across nearly all types of cancer research. Limited trust has been previously linked to reduced participation in clinical trials and data sharing.^5^^,^^6^ The magnitude of these associations is particularly concerning for research activities that are essential to advancing cancer prevention and treatment, such as clinical trials, genetic testing, and biospecimen collection. These patterns suggest that trust functions as a foundational determinant of research engagement, influencing participation even in activities with relatively low burden. As cancer research increasingly relies on broad public involvement, understanding and addressing trust deficits will be critical for ensuring equitable participation and scientific progress.

A key limitation of this study is its reliance on self-reported survey data, which may introduce reporting biases and may not fully capture actual behavior in real-world research settings. Findings of this survey may not be generalizable broadly, as the survey was restricted to SC only. Additionally, the cross-sectional design limits causal inference regarding the directionality of trust and willingness to participate. However, the study has several strengths, including a large sample, use of a pre-validated instrument, and assessment of willingness to engage across a broad range of cancer research activities.

In conclusion, the results of this study highlight that limited public trust in researchers may hinder recruitment and reduce the representativeness of cancer research, with downstream consequences for population-level cancer prevention and control efforts. Because public participation is foundational to generating evidence that informs cancer screening guidelines, treatment innovations, and survivorship care, a lack of trust in researchers poses a significant threat to public health. Targeted trust-building strategies are urgently needed to strengthen public participation to ensure the generalizability of cancer research.

## Supplementary Material

oyag170_Supplementary_Data

## Data Availability

De-identified data is available via MUSC SHARE upon IRB and research protocol approval.
