# Supplementary material for: Trust in researchers and willingness to engage in cancer research
Source: Oncologist. 2026 Apr 30;31(6):oyag170. doi: 10.1093/oncolo/oyag170 (PMC13157339; doi:10.1093/oncolo/oyag170)
Supplement: oyag170_Supplementary_Data [file oyag170_supplementary_data.docx]

**Supplementary Material**

**Trust in Researchers and Willingness to Engage in Cancer Research**

Kalyani Sonawane, PhD, Alexander Alekseyenko, PhD, Gayenell Magwood, PhD, RN, Shikhar Mehrotra, PhD, Gayathri Devi, PhD, MS, Marvella E Ford, PhD

**STable 1:** Trust in Medical Researchers (TMRS) 12-Item survey instrument.

On a scale from 1 to 5, with 1 being strongly disagree and 5 being strongly agree, how much do you agree with each statement? *Select one response per statement.*

| **TMRS Item** | **Strongly**  **Disagree** | **Disagree** | **Neutral** | **Agree** | **Strongly**  **Agree** |
| --- | --- | --- | --- | --- | --- |
| #1 To get people to take part in a study, cancer researchers usually do not explain all of the dangers about participation. | 1 | 2 | 3 | 4 | 5 |
| #2 Participants should be concerned about being deceived or misled by cancer researchers. | 1 | 2 | 3 | 4 | 5 |
| #3 Usually, researchers who make mistakes try to cover them up. | 1 | 2 | 3 | 4 | 5 |
| #4 Cancer researchers act differently toward minority subjects than toward white subjects. | 1 | 2 | 3 | 4 | 5 |
| #5 Cancer researchers unfairly select minorities for their most dangerous research studies. | 1 | 2 | 3 | 4 | 5 |
| #6 Some medical research projects are secretly designed to expose minority groups to diseases such as AIDS. | 1 | 2 | 3 | 4 | 5 |
| #7 Cancer researchers are generally honest in telling participants about different treatment options available for their conditions. | 1 | 2 | 3 | 4 | 5 |
| #8 Usually, cancer researchers tell participants everything about possible dangers. | 1 | 2 | 3 | 4 | 5 |
| #9 All in all, cancer researchers would not conduct experiments on people without their knowledge. | 1 | 2 | 3 | 4 | 5 |
| #10 Most cancer researchers would not lie to people to try to convince them to participate in a research study. | 1 | 2 | 3 | 4 | 5 |
| #11 In general, medical researchers care more about doing their research than about the participants’ medical needs. | 1 | 2 | 3 | 4 | 5 |
| #12 Researchers are more interested in helping their careers than in learning about health and disease. | 1 | 2 | 3 | 4 | 5 |

**Scoring:** The scores from the 12 items are summed unweighted, and 12 is subtracted from the total. The total will range from 0-48, with the higher the score, the greater the trust in medical researchers. Items # 1-6 and 11-12 are reverse-coded.

**Adapted from:** Mainous AG 3rd, Smith DW, Geesey ME, Tilley BC. Development of a measure to assess patient trust in medical researchers. Ann Fam Med. 2006 May-Jun;4(3):247-52. doi: 10.1370/afm.541. PMID: 16735527; PMCID: PMC1479445.

**STable 2:** Survey items capturing willingness to engage in cancer research overall and by type of research activity.

Please indicate your attitude about participating in cancer research and clinical trials. On a scale from 1 to 4, with 1 being Not likely at all to participate and 4 being Likely, how much do you agree with the following statement.

|  | **Not likely at all to participate** | **Not likely** | **Somewhat likely** | **Likely** |
| --- | --- | --- | --- | --- |
| If you were approached by someone to participate in a study, how likely would you be to volunteer to participate in a study in the next 3 months? | 1 | 2 | 3 | 4 |

Which of the following potential cancer research opportunities would you be willing to participate in? *Check all that apply.*

| **I would be willing to participate in*:**  □ Research/Clinical trials  □ Community-based studies such as healthy eating, exercise, or tobacco cessation  □ Cancer screenings    □ Genetic testing _______________ |
| --- |

Please choose which of the below you would be willing to provide for the purpose of cancer research. *Check all that apply.*

| **As a patient, I would be willing to provide:**  □ Saliva (spit or cheek swab) samples for study □ Urine samples for study  □ Left over blood, tissue, or other fluids □ New blood samples for study  □ Stool samples for study □ Medical records or lab results |
| --- |

**Adapted from:** *National Cancer Institute: Health Information National Trends Survey. Available at: <https://hints.cancer.gov/> **Melikam, E. S., Magwood, G. S., Ford, M., Salley, J., Abraham-Hilaire, L., Nelson, J., McCrary-Quarles, A., Berry, C., & Cartmell, K. B. (2024). Community Trust, Attitudes and Preferences Related to Participation in Cancer Research in South Carolina. *Journal of community health*, *49*(1), 100–107. [https://doi.org/10.1007/s10900-023-01251-3](https://urldefense.com/v3/__https:/doi.org/10.1007/s10900-023-01251-3__;!!Ab1_Rw!GyMGWduRWTtWdmOyRnLjAEnPqcEWOZIEq4Y8mbgEPKhbCjvizsyOekBzG67LTVDrNRIvyvLiHnE5_O2M$)
